# Supplementary material for: Splice-Junction-Based Mapping of Alternative Isoforms in the Human Proteome
Source: Cell Rep. Author manuscript; Available in PMC 2020 Jan 15. (PMC6961840; doi:10.1016/j.celrep.2019.11.026)
Supplement: 2 [file NIHMS1546469-supplement-2.zip › DF1/PXD010154/Thyroid/Thyroid_37_TG_VTGGQPACESVTDCQRNEAGLQCDQNGQYR.pdf]

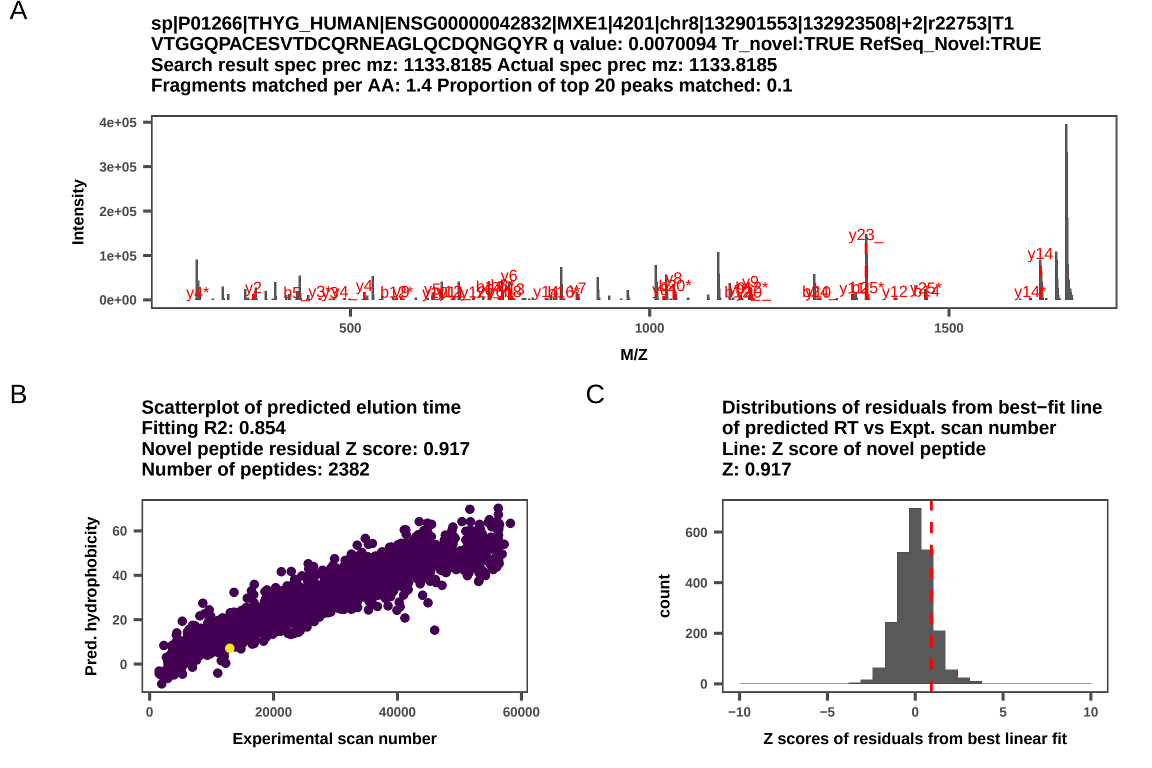

A
sp|P01266|THYG_HUMAN|ENSG00000042832|MXE1|4201|chr8|132901553|132923508|+2|r22753|T1
VTGGQPACESVTDCQRNEAGLQCDQNGQYR q value: 0.0070094 Tr_novel:TRUE RefSeq_Novel:TRUE
Search result spec prec mz: 1133.8185 Actual spec prec mz: 1133.8185
Fragments matched per AA: 1.4 Proportion of top 20 peaks matched: 0.1
Intensity
4e+05
3e+05
2e+05
1e+05
0e+00
y23_
y14
y6
y8
y9
y7
y2
y4
y4*
y14*
M/Z
500
1000
1500
B
Scatterplot of predicted elution time
Fitting R2: 0.854
Novel peptide residual Z score: 0.917
Number of peptides: 2382
Pred. hydrophobicity
60
40
20
0
Experimental scan number
0
20000
40000
60000
C
Distributions of residuals from best−fit line
of predicted RT vs Expt. scan number
Line: Z score of novel peptide
Z: 0.917
count
600
400
200
0
Z scores of residuals from best linear fit
−10
−5
0
5
10
